# Pharmaceutical care of chloroquine phosphate in elderly patients with coronavirus pneumonia (COVID‐19)

**DOI:** 10.1002/agm2.12104

**Published:** 2020-04-06

**Authors:** Xuelin Sun, Shaoqiang Li, Kexin Li, Xin Hu

**Affiliations:** ^1^ Department of Pharmacy Beijing Hospital National Center of Gerontology Institute of Geriatric Medicine Assessment of Clinical Drugs Risk and Individual Application Key Laboratory Chinese Academy of Medical Sciences Beijing China; ^2^ Department of Pharmacy Administration and Clinical Pharmacy School of Pharmaceutical Sciences Peking University Beijing China; ^3^ Clinical Trial Center Beijing Hospital National Center of Gerontology Institute of Geriatric Medicine Assessment of Clinical Drugs Risk and Individual Application Key Laboratory Chinese Academy of Medical Sciences Beijing China

## 1 INTRODUCTION

Since the outbreak of the new coronavirus pneumonia (COVID‐19) in December 2019, caused by severe acute respiratory syndrome coronavirus 2 (SARS‐CoV‐2), it has spread to the whole country. By 12:00 am on February 28, 2020, a total of 78 632 cases had been confirmed nationwide, including 2747 deaths. At present, it has been found that people of all ages are generally susceptible to the new virus, but the elderly and patients with multiple chronic diseases have the highest rates of severe illness and mortality.^1^, ^2^ According to the COVID‐19 development and treatment of the epidemic situation, the state has formulated and updated six versions of the pneumonia diagnosis and treatment plan for COVID‐19. In the newly released “New Coronavirus Pneumonia Diagnosis and Treatment Plan (Trial Version 6)” by the National Health Commission of the People's Republic of China, chloroquine phosphate was officially recommended in the antiviral treatment.^3^ The recommended use of this drug is chloroquine phosphate 500 mg/time, bid, with a course of treatment not exceeding 10 days. After thorough discussion by the Guangdong Provincial Department of Science and Technology and the Guangdong Provincial Health Commission on Chloroquine Treatment of COVID‐19 multicenter cooperative group, chloroquine tablets were approved for use at a dose of 500 mg, bid, for a course of 10 days after chloroquine contraindications have been excluded for patients diagnosed as having mild, common, and severe cases of COVID‐19.^4^ In order to ensure that the drug is safe and effective in clinical practice, the National Health Commission of the People’s Republic of China and the National Administration of Traditional Chinese Medicine jointly issued the notice on adjusting the usage and dosage of chloroquine phosphate in the treatment of COVID‐19 to apply to adults aged 18‐65 years: patients with a bodyweight of 50 kg or above, 500 mg, bid, for 7 days; and those with a bodyweight of 50 kg or less, 500 mg, bid, on the 1st and 2nd days, and 500 mg, qd, on the 3rd to 7th days, with a course of treatment of 7 days.

Chloroquine phosphate is an antimalarial drug that has been on the market for many years. In 1820, the famous French pharmacists Pelletier and Caventou successfully extracted the earliest pure antimalarial drug, quinine (also known as cinchona), from cinchona bark. German scientist Andersag was the first to synthesize chloroquine in 1934, which caused serious adverse drug reactions (“cinchona reaction”) to some users due to excessive dosage of chloroquine. In 1944, scientists modified the structure of chloroquine by adding a hydroxyl group to it, and developed a new antimalarial drug, hydroxychloroquine, which was less toxic and more effective against malaria.^5^

At present, two independent experiment teams from China have confirmed that chloroquine has an impact against the SARS virus in vitro.^6^, ^7^ Joint research results from the Wuhan Institute of Virology, Chinese Academy of Sciences and the Beijing Institute of Pharmacology and Toxicology showed that remdesivir (GS‐5734) and chloroquine (Sigma, C6628) effectively inhibit the recently emerged SARS‐CoV‐2 in vitro.^8^ Based on these results, clinical trials of chloroquine were conducted in hospitals in Beijing and Guangdong province, and the results showed that chloroquine phosphate may be effective in treating COVID‐19.

At present, the safety of chloroquine phosphate in the treatment of elderly patients with COVID‐19 cannot be determined, but the rate of critical illness in the elderly is high and chloroquine phosphate can still be used as an alternative antiviral drug for the elderly. The use of chloroquine phosphate is prohibited in the following elderly patients: those who are clearly allergic to 4‐aminoquinoline compounds; those with arrhythmia (eg, conduction block) or chronic heart disease; those with chronic liver and kidney diseases who are reaching the terminal stage; those with known retinal disease or hearing loss; those with known psychiatric disorders; those with skin diseases (including rashes, dermatitis, and psoriasis); and those with glucose‐6‐phosphate dehydrogenase deficiency.

## 2 CHLOROQUINE PHOSPHATE FOR TREATMENT OF ELDERLY WITH COVID‐19

Based on the published clinical guidelines and research results, this paper proposes the following pharmaceutical care for the elderly using chloroquine phosphate in the treatment of COVID‐19.

### 2.1 Administration method of chloroquine phosphate

Oral administration of chloroquine phosphate is the only suggested administration in elderly patients.

### 2.2 Dosage of chloroquine phosphate

For elderly patients with a bodyweight of more than 50 kg, chloroquine phosphate 500 mg orally, bid, for 7 days is recommended. For those with a bodyweight of 50 kg or less, 500 mg, bid, on the 1st and 2nd days, and 500 mg, qd, on the 3rd to 7th days, with a course of treatment of 7 days is recommended. According to the Wuhan Institute of Virology, Chinese Academy of Sciences, the lethal dose of chloroquine phosphate in adults is 2‐4 g and doses should be closely monitored during the treatment period. The concentration of chloroquine phosphate is maintained for a long time, the plasma protein‐binding rate is about 55%, and the half‐life is 2.5‐10 days. The drug is metabolized by the liver, and 10%‐15% of the drug is excreted by the kidney as the original. Older patients with impaired kidney function may be at greater risk for toxic reactions to the drug. Elderly patients are more likely to suffer from decreased renal function, decreased metabolism of drugs, and weakened excretory function. Therefore, the drug residual dose in patients taking chloroquine phosphate for 7 consecutive days may reach or even exceed the lethal dose.^9^

Oral chloroquine can be quickly absorbed. Elderly patients should be advised to take it with food. Serious toxic reactions can occur within 1 to 3 hours, or even a few minutes after use. Acute poisoning can be fatal, with a lethal dose as low as 50 mg/kg. Death can occur within 2 to 3 hours. Once the above symptoms appear, the medication should be discontinued immediately and symptomatic treatment should be carried out, especially to maintain cardiopulmonary function. Refer to the treatment advice for acute hydroxychloroquine poisoning: induce vomiting or emptying of the stomach contents as soon as possible; within half an hour of taking the medicine, you can take activated carbon powder to inhibit further absorption of the drug, and use angiotensin drugs when shock occurs; provide fluid replacement at the same time; and provide plenty of ammonium chloride (8 g/day) to promote the excretion of the drugs. Acute poisoning survivors should be closely monitored for at least 6 hours, even if they show no symptoms.

### 2.3 Adverse drug reactions of chloroquine phosphate

The main adverse reactions of chloroquine phosphate are as follows:

1. Cardiovascular system: sinus node inhibition, arrhythmia, and severe occurrence of Adams‐Strokes syndrome.
2. Skeletal musculoskeletal system: neuromuscular pain.
3. Nerves: irritability, medicated psychosis.
4. Blood system: granulocytopenia, aplastic anemia, thrombocytopenia.
5. Eye: irreversible visual impairment.
6. Skin: itching, rash, purpura, dermatitis, etc.
7. Ears: tinnitus, hearing impairment.
8. Gastrointestinal symptoms: loss of appetite, nausea, vomiting, diarrhea, and abdominal pain.

Adverse drug reactions to chloroquine phosphate involve almost every system and may be more severe in the elderly. According to the 2013 FDA adverse reactions advisory, chloroquine phosphate may cause macular degeneration and severe extrapyramidal disease, which may occur more frequently in the elderly and should be carefully monitored during use. Close attention should be paid to: 

1. Blood routine examination. Blood routine monitoring should be conducted closely every other day during the medication period. If white blood cells progressively decrease or anemia and thrombocytopenia progressively increase, the dosage should be reduced or discontinued. 

2. Routine electrocardiogram examination should be conducted before treatment and the electrocardiogram should be monitored at Days 1, 3, 5, and 7 of treatment. In case of serious adverse reactions, such as Q‐T interval extension, atrioventricular block and torsional tachycardia, the prescription should be stopped immediately. 

3. Check the patient’s vision changes regularly. If the elderly also suffer from other eye diseases, regular eye examination is needed to prevent the occurrence of retinopathy and serious clinical manifestations of the main “target‐center eye.” Stop the medicine immediately in case of such poisoning. 

4. Regularly check the patient’s ankle and knee reflexes in case the medicine causes skeletal muscle injury. Elderly patients often spend a long time in bed, which may mask such symptoms, so the examination process should be especially careful. 

5. Pay attention to the mental and psychological state of elderly patients. In the isolation process of treatment, patients with neopneumonia may suffer from excessive pressure, resulting in forgetfulness, insomnia, depression, and so forth, so attention should be paid to distinguish these symptoms from the adverse reactions caused by chloroquine.

### 2.4 Drug interactions of chloroquine phosphate

According to the drug instructions of chloroquine phosphate, it can interact with the following drugs, which may lead to adverse reactions and adverse events when used together.

1. Chloroquine (hydroxychloroquine): causes increased concentration of chloroquine in blood.
2. Chlorpromazine: combined use increases liver burden.
3. Monoamine oxidase inhibitors: combined use increases toxicity.
4. Heparin and penicillamine: combined use increases the risk of bleeding.
5. Digitalis: the use of this drug after digitalis causes heart block.
6. Ammonium chloride: used in combination to aggravate the excretion of this medicine.
7. Streptomycin: the combination of this drug increases the direct inhibitory effect on the neuromuscular junction.
8. Butazone: combination often causes allergic dermatitis.
9. Triamcinolone: combined use leads to Exfoliative erythroderma.
10. Nifedipine: combined use leads to increased blood concentration of chloroquine.

Due to the interaction between chloroquine phosphate and the above drugs, clinicians of elderly patients should pay close attention to the combined use in the treatment process. Great care should be taken if elderly patients are taking chloroquine phosphate in combination with the following drugs due to illness: digitalis drugs, bute, heparin, penicillamine, amiodarone, benzyl, general, domperidone and droperidol, haloperidol, azithromycin, astemizole, erythromycin, clarithromycin, posaconazole, methadone, procainamide, hydrochlorothiazide, spa, levofloxacin, moxifloxacin, cisapride, indapamide, chlorpromazine, streptomycin, ammonium chloride, ondansetron, apomorphine, octreotide, monoamine oxidase inhibitors, and fluorine hydroxyl prednisolone.

## 3 CONCLUSIONS

According to current reports, the population is generally susceptible to SARS‐CoV‐2 and older people with underlying diseases are more likely to develop severe illness. Cardiac arrest is the most serious adverse reaction of chloroquine phosphate. Elderly patients who are prescribed chloroquine phosphate for treatment of COVID‐19 must have a normal electrocardiogram before medication. Chloroquine phosphate is prohibited in combination with drugs that cause Q‐T prolongation, such as quinolones and macrolides. At the same time, the electrolyte level (potassium, sodium, chlorine), blood glucose, and liver and kidney functions of patients should be confirmed as normal. Elderly patients should not be treated with three or more antiviral drugs, including chloroquine phosphate. Medical staff must pay close attention to adverse reactions after medication, and should cease medication when there are intolerable side‐effects. Furthermore, staff should monitor and report adverse drug reactions in strict accordance with the requirements of the measures for the reporting, monitoring, and management of adverse drug reactions, so as to ensure drug safety for the elderly during the treatment of COVID‐19.

## CONFLICTS OF INTEREST

Nothing to disclose.

## AUTHOR CONTRIBUTIONS

Xuelin Sun and Xin Hu conceptualized the study. Xuelin Sun wrote the original draft of the manuscript. Shaoqiang Li provided methodology. Xin Hu provided data resources and supervised the study. Kexin Li reviewed and edited the manuscript.
